# Macular Thickness Decrease in Asymptomatic Subjects at High Genetic Risk of Developing Alzheimer’s Disease: An OCT Study

**DOI:** 10.3390/jcm9061728

**Published:** 2020-06-03

**Authors:** Inés López-Cuenca, Rosa de Hoz, Elena Salobrar-García, Lorena Elvira-Hurtado, Pilar Rojas, José A. Fernández-Albarral, Ana Barabash, Juan J. Salazar, Ana I. Ramírez, José M. Ramírez

**Affiliations:** 1 Instituto de Investigaciones Oftalmológicas Ramón Castroviejo, Universidad Complutense de Madrid (UCM), 28040 Madrid, Spain; inelopez@ucm.es (I.L.-C.); rdehoz@med.ucm.es (R.d.H.); elenasalobrar@med.ucm.es (E.S.-G.); marelvir@ucm.es (L.E.-H.); pilar.rojas.lozano@gmail.com (P.R.); joseaf08@ucm.es (J.A.F.-A.); jjsalazar@med.ucm.es (J.J.S.); 2 IIORC, Faculty of Medicine, 28011 Madrid, Spain; 3 Facultad de Óptica y Optometría, Departamento de Inmunología, Oftalmología y ORL, UCM, 28037 Madrid, Spain; 4 Hospital General Universitario Gregorio Marañón, Instituto Oftálmico de Madrid, 28007 Madrid, Spain; 5 Endocrinology and Nutrition Department, Hospital Clínico Universitario San Carlos and Instituto de Investigación Sanitaria del Hospital Clínico San Carlos, 28040 Madrid, Spain; ana.barabash@gmail.com; 6 Centro de Investigación Biomédica en Red de Diabetes y Enfermedades Metabólicas Asociadas, 28029 Madrid, Spain; 7 Facultad de Medicina, Departamento de Inmunología, Oftalmología y ORL, UCM, 28040 Madrid, Spain

**Keywords:** Alzheimer´s disease, ApoE ɛ4, first-degree family history, genetic risk, OCT, retina

## Abstract

In this case control study, we examined the retinal thickness of the different layers in the macular region and peripapillary retinal nerve fiber layer (RNFL) with optical coherence tomography (OCT) in healthy cognitive subjects (from 51 to 74 years old) at high genetic risk for developing Alzheimer’s disease (AD). Thirty-five subjects with a family history of Alzheimer disease (AD) (FH+) and ApoE ɛ4 carriers and 29 age-matched control subjects without a family history of AD (FH−) and ApoE ɛ4 non-carriers were included. Compared to FH− ApoE ɛ4 non-carriers, in FH+ ApoE ɛ4 carriers, there were statistically significant decreases (*p* < 0.05) in (i) the foveal area of mRNFL; (ii) the inferior and nasal sectors in the outer and inner macular ring in the inner plexiform layer (IPL); (iii) the foveal area and the inferior sector in the outer macular ring in the inner nuclear layer (INL); and (iv) the inferior sector of the outer macular ring in the outer plexiform layer (OPL). However, no statistically significant differences were found in the peripapillary thickness of RNFL between both study groups. In subjects with cognitive health and high genetic risk for the development of AD, initial changes appeared in the macular area. OCT could be a promising, cost-effective and non-invasive test useful in early AD, before the onset of clinical symptoms.

## 1. Introduction

One of the main genetic risk factors for developing sporadic Alzheimer’s Disease (AD) is having a first-degree family history of the disease [1], another major genetic risk factor is mediated by ApoE [2]. This gene has been implicated in modulating the metabolism and aggregation of Aβ [3]. There are three alleles of APOE, ɛ2, ɛ3 and ɛ4, and they encode for apolipoprotein E (ApoE) [4]. ApoE4 increases the risk of AD by three times per ɛ4 allele, with the risk being highest among people of European descent [5]. Moreover, the mean age at the clinical onset of AD has been estimated at 68 years for ɛ4 homozygotes and 76 years for ɛ4 heterozygotes [6]. Although genetics is not included in the research framework because the gene variants do not measure pathological changes or indicate any particular stage of AD [7], ApoE ɛ4 has been reported to affect magnetic resonance imaging (MRI), cerebrospinal fluid (CSF) and cognitive biomarkers [8], and it may have intrinsic effects on brain function [9]. In addition, ApoE ɛ4 plus high levels of amyloid beta (Aβ) proteins are associated with episodic memory decline and high risk for clinical AD [10], and ApoE ɛ4 carriers are also more vulnerable to environmental factors [11].

For years, changes in subjects at high risk of developing AD have been studied [12,13,14,15,16]. Casual findings such as the presence of white matter hyper-intensities, vascular abnormalities and the loss of brain volume have been reported in some MRI studies [12,17]. In magnetoencephalographic (MEG) studies, the functional connectivity of cognitively healthy subjects at high risk of AD showed areas where this connectivity decreased and others where it improved. In these areas between the precuneus and the bilateral inferior parietal lobes, in which there is more activity, they have a neuronal hyper-synchronization that can be explained as an early abnormal excitatory response by the effect of Aβ accumulation [18]. Previously, it has been reported that neurons near amyloid plaques become hyperactive [19], showing a decrease in synaptic inhibition as a possible cause of hyperactivity. It is known that signs of dementia can appear decades before clinically detectable symptoms [20,21]. For all these reasons, interest in finding new biomarkers of the disease has focused on earlier stages of AD [22], searching for less invasive methods and easily accessible tissues such as the retina [23,24].

There is a clear link between retinal and cerebral changes in AD [23]. In preclinical and prodromal stages, retinal changes have also been reported in the optical coherence tomography (OCT) [25,26], as well as the existence of associations between quadrant-specific retinal nerve fiber layer (RNFL) thickness and the brain regions analyzed by 3T MRI [27].

The aim of this work was to analyze by OCT the existence of possible structural changes in the retina, both in the macular and in the peripapillary area, in subjects with two marked genetic risk factors for AD: first-degree family history of AD and being carrier of at least one ɛ4 allele for the ApoE gene.

## 2. Materials and Methods

### 2.1. Study Design

“The cognitive and neurophysiological characteristics of subjects at high risk of developing dementia: a multidimensional approach” (COGDEM study) was conducted in different centers including the Centre for Biomedical Technology (CBT), the San Carlos Clinical Hospital in Madrid (HCSC), the Ramon Castroviejo Institute of Ophthalmic Research (IIORC) in the Complutense University of Madrid (UCM), Spain. All the study participants provided written informed consent. The research followed the tenets of the Declaration of Helsinki, and the study was approved by the local Ethics Committee (HCSC) with the internal code 18/422-E_BS.

We analyzed two groups: subjects with a family history of AD (FH+) and their respective controls. The group (FH+) was made up of middle-aged subjects with at least one parent with sporadic AD. Their clinical records should not show a history of neurological or psychiatric disorders or suffering from a serious medical condition. To verify the parent’s diagnosis of AD, a multidisciplinary diagnostic consensus panel reviewed the parent’s medical records. Experts at the HCSC reviewed the diagnostic procedures to select only those diagnoses that were made under internationally accepted criteria. Although autopsy reports verified by a pathologist were welcome, this may not be an inclusion criterion since this procedure was not performed in most AD patients. Families with known autosomal dominant mutations (i.e., preseniline-1 or 2) were not included. As controls for the FH+ group, we selected middle-aged subjects in whom the absence of a first-degree family history of AD (FH−) was determined through the participant’s self-report in response to a detailed medical history questionnaire. This FH− group was matched with the FH+ group in terms of age, socioeconomic status and other demographic characteristics. In addition, no previous history of neurological or psychiatric disorder or any serious medical condition was an inclusion criterion for this sample. Both the FH+ and FH− groups showed normal scores (above 26) on the Mini Mental State Examination (MMSE). They also showed normal MRI with no evidence of brain lesion or pathology.

Because approximately 15% of the general population carries ApoE ɛ4, we decided to include this genetic factor as a covariate in our study. For the analysis of ApoE genotyping, DNA was extracted from 10 mL blood samples in ethylenediaminetetraacetic acid from the FH+ and FH− subjects. ApoE was detected using TaqMan assay technology on an Applied Biosystems 7500 Fast Real-Time PCR machine (Applied Biosystems, Foster City, CA, USA). ApoE alleles were determined by analyzing two single-nucleotide polymorphisms (SNPs), rs7412 and rs429358 genotypes, with TaqMan Genotyping Assays (C____904973_10 and C___3084793_20, respectively).

### 2.2. Subjects

We reviewed the COGDEM study database, which consists of 251 patients. In our study, none of the subjects could have an ophthalmological pathology that could modify the measures by OCT, and for this reason we performed a phone screening with all the patients to assess their ophthalmological history. The questions are shown in Table 1. Forty-four subjects refused to participate, and seventeen had ocular pathology.

### 2.3. Ophthalmological Tests

All remaining participants (*n* = 190) were examined in the clinic of the Ramon Castroviejo Institute of Ophthalmic Research, where an eye examination was performed. For the study, one eye of each patient was randomly selected. The visual exam included visual acuity, slit-lamp examination, applanation tonometry (Perkins MKII tonometer, Clement Clarke International, Essex, England), a dilated fundus and an OCT examination (Heidelberg Engineering, Heidelberg, Germany).

In addition to the general inclusion criteria, all the participants met the ophthalmological inclusion criteria listed in Table 2.

**Figure 1:**
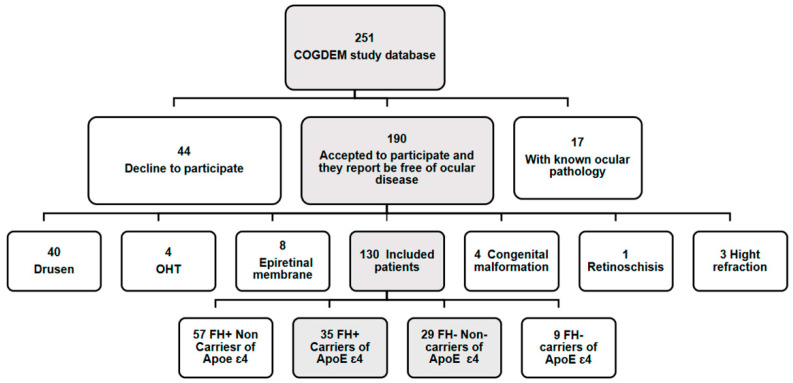
Flow diagram of patient’s inclusion. (COGDEM): “The cognitive and neurophysiological characteristics of subjects at high risk of developing dementia: a multidimensional approach”; (FH+), subjects with a family history of Alzheimer’s disease (AD); (FH−) subjects without a family history of AD. In greyscale, patients who participated in the ophthalmological study and were included in our study.

Of these 190 subjects, after the ophthalmological examination, we excluded 60 participants for different ophthalmologic pathologies or conditions that were unknown for them. Finally, the remaining 130 patients were divided into 57 FH+ ApoE ɛ4 non-carriers, 35 FH+ ApoE ɛ4 carriers, 29 FH− ApoE ɛ4 non-carriers and 9 FH− ApoE ɛ4 carriers. Since in our study we considered being an ApoE ɛ4 carrier as inclusion criteria, 35 FH+ ApoE ɛ4 carriers and 29 FH− ApoE ɛ4 non-carriers (control group) participated, being a total of 64 adults aged between 51 and 74 years (Figure 1).

### 2.4. Optical Coherence Tomography

OCT images were captured with Spectralis OCT (Heidelberg Engineering, Heidelberg, Germany). Spectralis Glaucoma Module Premium Edition was used, which offers an anatomic positioning system (APS) based on two fixed points: the center of the fovea and the center of Bruch’s membrane opening (BMO). At the optic nerve head (ONH), a 4-line high-resolution radial scan and three-circle scans, both centered on BMO, were acquired to provide highly reproducible RNFL thickness results. In the macular area, which was centered on the APS of Glaucoma Module Premium, 121 dense B-scans were performed per eye.

**Figure 2:**
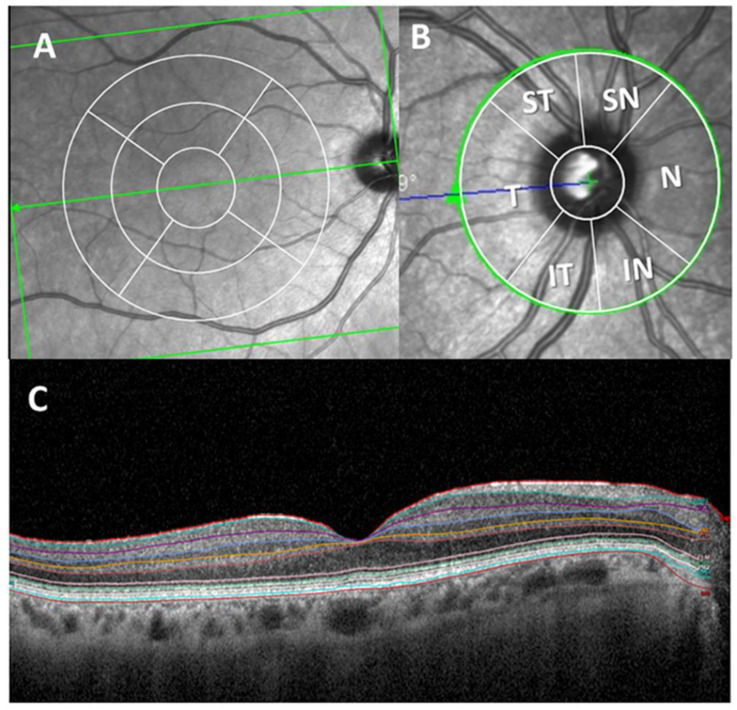
Optical coherence tomography (OCT) report of the retinal thickness analysis. (**A**) Concentric macular rings. (**B**) Peripapillary sectors. (**C**) Macular thickness segmentation of all retinal layers. (ST: supero-temporal; SN: supero-nasal; N: nasal; IN: infero-nasa; IT: infero-temporal; T: temporal and G: global).

The total thickness of the retina and the thickness of each retinal layer in the macular area were measured with Heildelberg segmentation software (Heidelberg, Germany, version 1.10.4.0). This segmentation was checked by the same optometrist (IL-C) and modified manually if required. The thicknesses of the following layers of the retina were analyzed: retinal nerve fiber layer (RNFL), ganglion cell layer (GCL), inner plexiform layer (IPL), inner nuclear layer (INL), outer plexiform layer (OPL), outer nuclear layer (ONL) and retinal pigment epithelium (RPE) (Figure 2C). The inner and outer macular rings were analyzed according to the standard Early Treatment Diabetic Retinopathy Study (ETDRS) macular grid (a foveal area of 1 mm of diameter, 1–3 mm around the fovea in the inner ring and 3–6 m for the outer ring) [28] (Figure 2A). Peripapillary RNFL (pRNFL) thickness was measured in six sectors (nasal, supero-nasal, infero- nasal, temporal, supero-temporal, infero-temporal), also obtaining an average over all sectors (Global) (Figure 2B). Good-quality scans were considered with a signal-to-noise ratio >30 and 95% accepted A-Scans. According to the calibration provided by the manufacturers, the measurements were given in microns. Both eyes of each subject were scanned, but only one eye of each participant was used in this study. The scans were obtained after pharmacological mydriasis at under room light conditions.

### 2.5. Statistical Analysis

Statistical analysis was carried out in SPSS 25.0 (SPSS Inc., Inc., Chicago, IL, USA). The distribution of the sample was analyzed using visual methods including histograms, Q-Q plot graphics as well as Kolmogorov–Smirnov and Shapiro–Wilk normality tests.

Data are reported as the median (interquartile range). The differences between the study groups (FH+ ApoE ɛ4 carriers and FH− ApoE ɛ4 non-carriers) were analyzed using a Mann–Whitney U Test. For the analysis of qualitative variables, a chi-squared test was used. A *p* value ≤ 0.05 was considered statistically significant.

## 3. Results

Demographic data for FH+ ApoE ɛ4 carriers and FH− ApoE ɛ4 non-carriers are shown in Table 3.

There were statistically significant differences (*p* < 0.05) between the sex of the subjects in the group of FH+ ApoE ɛ4 carriers (11 males/24 females), while in the control group there were no significant differences. Regarding the mean age of the participants, there was no significant difference between FH+ ApoE ɛ4 carriers (57.00 (54.00–61.00)) and FH− ApoE ɛ4 non-carriers (59.00 (54.00–65.00)). The mean MMSE scores were (29.00 (29.00–29.00)) in the FH+ ApoE ɛ4 carrier group and (29.00 (28.00–29.00)) in the FH− ApoE ɛ4 non-carrier group.

### 3.1. Macular Thickness Analysis by OCT

**Figure 3:**
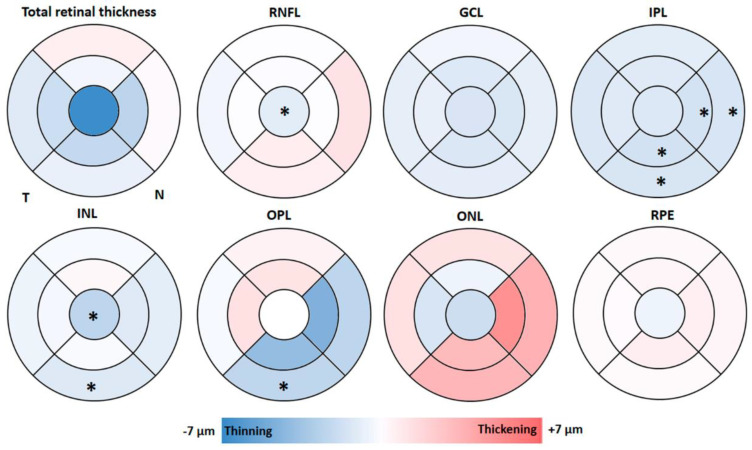
Colorimetric differences in the retinal thickness in each layer between the groups. FH+ ApoE ɛ4 carriers vs. FH− ApoE ɛ4 no-carriers in the macular OCT rings. In red, thickening; in blue, thinning. (RNFL: retinal nerve fiber layer; GCL: ganglion cell layer; IPL: inner plexiform layer; INL: inner nuclear layer; OPL: outer plexiform layer; ONL: outer nuclear layer; RPE: retinal pigment epithelium). * *p* < 0.05. Mann–Whitney U test.

Regarding the total retinal thickness, there were no significant differences between the study groups in any of the sectors analyzed (*p* > 0.05), with the foveal sector being the thinnest in the eyes of FH+ ApoE ɛ4 carriers in comparison to eyes of FH− ApoE ɛ4 non-carriers (Figure 3).

The thickness of the macular retinal nerve fiber layer (mRNFL) showed a significant thinning (*p* < 0.05) in the foveal area in the group of FH+ ApoE ɛ4 carriers (12.00 (10.00–13.00)) compared to that of the group of FH− ApoE ɛ4 non-carriers (13.00 (11.50–14.00)). (Figure 3).

In comparison to the FH− ApoE ɛ4 non-carrier group, FH+ ApoE ɛ4 carriers showed a non-significant, slight reduction of all the sectors in the thickness of GCL (Figure 3).

In the IPL of FH+ ApoE ɛ4 carriers, the inferior sectors, both in the inner macular ring (41.00 (39.00–42.00)) and outer macular ring (27.00 (25.00–29.00)), were statistically reduced (*p* < 0.05 in both cases) with respect to the FH− ApoE ɛ4 non-carrier group. This significant thinning (*p* < 0.05) was also observed in the inner (42.00 (40.00–44.00)) and outer (30.00 (27.00–31.00)) macular rings in the nasal sector. (Figure 3).

In the INL, the FH+ ApoE ɛ4 carriers, with respect to the FH− ApoE ɛ4 non-carrier group, showed a significant thickness decrease in the outer macular ring in the inferior sector (31.00(29.00–32.00)) and in the foveal sector (18.00 (16.00–21.25)) (*p* < 0.05 in both cases) (Figure 3).

In the OPL of the FH+ ApoE ɛ4 carriers, a significant decrease (*p* < 0.05) in the outer macular ring in the inferior sector (27.00 (26.00–29.00)) was observed compared to the group of FH− ApoE ɛ4 non-carriers (30.00 (27.00–32.50)). (Figure 3).

In the analysis of ONL, no significant difference was observed between the two study groups. However, in FH+ ApoE ɛ4 carriers, with respect to FH− ApoE ɛ4 non-carriers, a slight increase in the thickness of the outer macular ring was observed in all sectors, as well as in the nasal and inferior sectors of the inner macular ring (Figure 3).

Regarding the thickness of the RPE, there were no statistically significant differences between the two study groups in any of the analyzed sectors (Figure 3).

### 3.2. Peripapillary RNFL Segmentation Thickness Analysis by OCT

**Figure 4:**
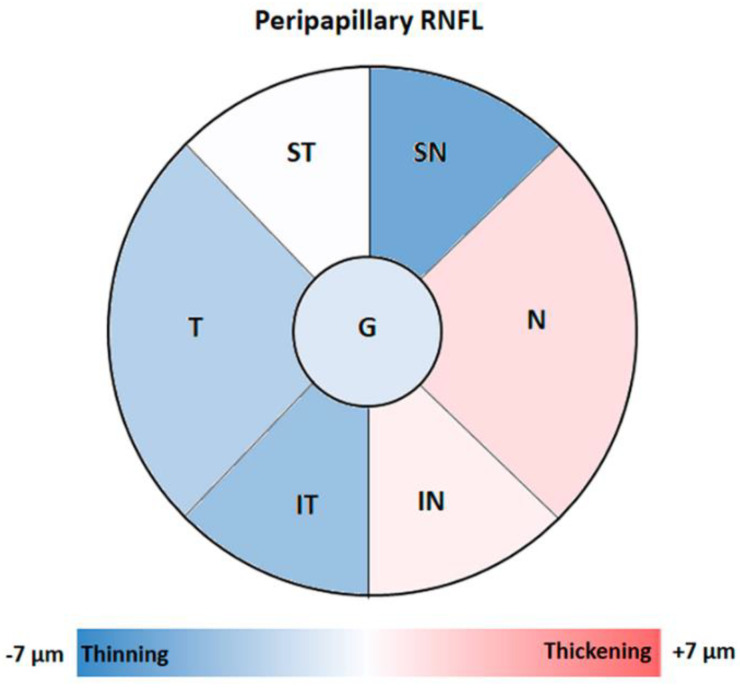
Colorimetric differences in the peripapillary RNFL thickness between the groups. FH+ ApoE ɛ4 carriers vs. FH− ApoE ɛ4 non-carriers in the peripapillary OCT sectors. In red, thickening; in blue, thinning. (ST: supero-temporal; SN: supero-nasal; N: nasal; IN: infero-nasal; IT: infero-temporal; T: temporal and G: global). Non-statistical differences were observed.

In the pRNFL, the comparison between the FH+ ApoE ɛ4 carriers with respect to the FH− ApoE ɛ4 non-carriers showed no statistically significant thickness differences in any of the sectors analyzed. However, in the FH+ ApoE ɛ4 carrier group, while a slight decrease was found in the superior nasal, inferior temporal and temporal sectors, a slight increase was observed in the nasal and nasal inferior sectors with respect to the FH− ApoE ɛ4 non-carrier group (Figure 4).

## 4. Discussion

To our knowledge, few studies have analyzed retinal thickness in healthy cognitive subjects at high genetic risk of developing Alzheimer’s dementia [25,26,29,30,31]. One of the highlights of this study was the careful selection of cases. All participants were free of ocular pathology and cognitive, psychological and behavioral disorders that could mask the results. Only family members of people diagnosed with AD were selected for this study.

In our study, in the FH+ ApoE ɛ4 carrier group there was a statistically significant difference between the sex of the participants (11 men/24 women). This difference could be explained by women being more likely to develop dementia, so they may be more interested in participating in the study [32]. In addition, women have traditionally been the caregivers because they may feel the losses associated with mental disorders in their families more acutely than men [33]. They commonly participate in studies for two reasons: advances in AD research that will benefit family, friends, or future generations and personal concerns about memory [34].

The association between pRNFL thickness measurements and brain structure volumes in non-demented older adults is now well known [27,35]. There are direct correlations between RNFL thickness in the temporal quadrant with medial temporal lobe volume and especially with the hippocampus volume [27]. Moreover, the inferior quadrant was associated with lingual gyrus volume. These changes, which were reported in previous studies, suggested that changes in the RNFL thickness could be used as an early marker of AD-related brain atrophy [27,36]. In addition, a decrease in pRNFL thickness has been correlated with impaired cognition during disease progression [37,38,39,40]. This pRNFL decrease was confirmed as a potential biomarker for predicting cognitive decline in older adults during preclinical dementia [36]. The association between the atrophy of brain areas and changes in retinal thickness means that the retina may be a potential biomarker for neurodegenerative diseases, as it is non-invasive, it is cost-effective and it is promising in the early stages of AD and even in subjects at high risk of developing dementia.

As far as we know, this is the first study that analyzed by OCT, both in macular and peripapillary regions, all the layers of the retina in cognitively healthy subjects at high risk of developing Alzheimer’s dementia due to two risk factors: having a family history and being a carrier of ApoE ɛ4. OCT was able to detect very early thickness changes in the macular region, which were very small (±7 µm) in comparison to thickness changes observed in diagnosed AD patients.

In our study, in the analysis of the mRNFL, we found a significant thinning in the foveal sector in the group of FH+ ApoE ɛ4 carriers compared to the FH− ApoE ɛ4 non-carrier group. It is surprising to find changes in retinal thickness among these study groups since it must be remembered that FH+ ApoE ɛ4 carriers are cognitively healthy subjects.

In preclinical studies of AD there are some differences. Some authors did not find significant differences through RNFL analysis in the total macular volume thickness between the AD preclinical group and the control group [29]. More recently, a significant decrease in the total macular volume has been described in preclinical AD with respect to the control groups. These changes appeared after a 27 month follow-up and were related to the neocortical accumulation of Aβ [26]. However, in another study, no significant differences were found between participants with Aβ deposits in the inner layers of the retina in the macular region and those without Aβ deposits in these layers [25]. In these three works, the preclinical groups were made up of people who had a family member with AD but also had subjective memory complaints. They were possibly in the pre-dementia stage with mild cognitive impairment (MCI), which the authors call the preclinical stage. In this stage, the pathology is present in the memory system of the medial temporal lobe, and the neurofibrillary pathology has already begun [26]. Aβ deposits could also be found in mRNFL, compensating in part for the decrease in mRNFL thickness seen in our FH+ ApoE ɛ4 carriers. Therefore, the reduction in mRNFL thickness could be observed before the damage to the mesiotemporal central nervous memory system, which is characteristic of early AD [26]. For this reason, we suggest that the loss of mRNFL thickness could be a feature of early AD or in subjects at high risk of developing AD.

In patients with established AD, a reduction in mRNFL thickness has been demonstrated in different sectors [37,41,42,43]. The difference between the inclusion criteria of these patients, in relation to the MMSE, makes the classification of the different stages of AD not equivalent. Possibly, for this reason, the results obtained by different authors were not the same. The greatest thinning was observed in the outer macular ring, in the superior and inferior sectors. This predominant alteration could be explained by the greater concentration of axonal bundles that were directed towards the optic nerve head in these sectors [23].

Few studies have analyzed the thickness of mRNFL in patients with MCI [36,44]. While some studies reported a reduction in the macular volume in MCI patients with respect to controls [36], others found increased macular volumes in MCI patients [44]. The increase of macular volume in MCI could be attributed to gliosis prior to neuronal loss and the atrophy of mRNFL [45].

In our study, the thickness of GCL in FH+ ApoE ɛ4 carriers showed a slight but not significant reduction in all the sectors with respect to controls. Most of the studies that analyzed this layer in preclinical AD groups also found no significant difference with respect to the control groups [25,26,29,30]. However, the decreased GCL thickness was the main cause of retinal thinning in patients diagnosed with AD [41]. This GCL thinning has been found both in mild AD patients [37] and mild-to-moderate AD patients [46]. In MCI, this thickness decrease has also been suggested as a possible tool to detect neuronal injury [47].

We found that the inferior and nasal sectors, both in the inner and outer macular rings of the IPL, showed significant thinning in the group of FH+ ApoE ɛ4 carriers compared to that of FH− ApoE ɛ4 non-carriers, with the IPL retinal layer showing more sectors with statistically significant thickness decreases. In a previous study, in subjects with subjective memory complaints, authors also found a significant thickness reduction in the inferior quadrant [26]. However, the results were different in patients with Aβ+ who showed an increase in the volume of this layer [30]. The thickness decrease found in our FH+ ApoE ɛ4 carrier subjects in IPL could be explained by the decreased cholinergic activity in this layer, as already described in histopathological studies of asymptomatic patients with the presence of Aβ deposits [48,49]. In more advanced stages of dementia (MCI and established AD), the GCL and the IPL have been analyzed as a complex, showing a thickness decrease in all the quadrants with respect to the control groups [41,47].

At INL, we found a significant thickness decrease in the foveal sector and in the inferior sector of the outer macular ring in our FH+ ApoE ɛ4 carriers. Only one previous study analyzed the thickness of all the retinal layers in the preclinical stage of AD, although they found no differences in the INL compared to the control group [26]. The absence of changes in this layer could be because this sample presented subjective memory complaints, as they were in a more advanced stage than our healthy cognitive subjects at high risk of developing dementia.

Regarding the outer layers of the retina, the inferior sector in the outer macular ring of the OPL was the only one with significant thinning. In addition, we found that the same sectors that showed a non-significant slight thickness decrease in the OPL (nasal and inferior) also showed a slight, non-significant increase in the ONL. In a previous study where we conducted a spatial analysis of thickness changes in the retinal layers of AD patients measured by OCT, we found that the use of concentric rings could detect the thickening and thinning of neighboring layers within the same region [50]. For this reason, the thickness changes of adjacent layers can be compensated for [50]. In addition, only in preclinical subjects who reported subjective memory complains were significant changes in ONL thickness detected, with respect to the control group, which were explained as a possible consequence of retrograde transynaptic degeneration [26].

Regarding RPE thickness, there were no statistically significant differences between our study groups in any of the sectors analyzed. No previous studies have analyzed this layer in preclinical patients. We found no significant changes in patients with established AD [37,51].

As expected, in the present work we did not find significant changes in any of the peripapillary sectors of RNFL. However, in patients with moderate AD we found a statistically significant thickness decrease in comparison to the control group. Changes in this peripapillary area have been associated with disease progression and the advance of cognitive decline, determined by the MMSE score [37].

Although we found an association between reduced retinal thickness in specific areas and the risk of developing AD dementia, a limitation of this study is that no data are available on how this thinning of the retina correlates with brain or retinal markers of AD. Although a family history of AD and an ApoE genotype ε4 increases the risk of developing AD dementia, not all of our patients at high risk of developing AD will develop the disease. Therefore, the high-risk group in this study possibly included both people who will develop AD dementia and some who will not. This could limit the ability of retinal thickness measurements in patients at high risk of developing AD to be used as an early biomarker. In addition, the same subjects would need to be analyzed over time to evaluate how many of them will develop the disease in the future. The subjects included in our study belonged to a multidimensional study (COGDEM) which will conduct their follow-up.

In conclusion, in cognitively healthy subjects who are asymptomatic but at high genetic risk of developing Alzheimer’s-type dementia, OCT is able to detect very early thickness changes in the macular region, which are very slight (around ± 7 µ). These changes showed statistically significant decreases in (i) the foveal area of the mRNFL; (ii) the inferior and nasal sectors in the outer and inner macular ring in the IPL; (iii) the foveal area and the inferior sector in the outer macular ring in the INL; and (iv) the inferior sector of the outer macular ring in the OPL. Therefore, OCT could be a useful tool for diagnosing, monitoring and screening patients at high genetic risk of developing AD, and it is a promising, cost-effective, non-invasive test useful for the early stages of the disease, before the onset of clinical symptoms.

## Figures and Tables

**Table 1:** Telephone screening questions.

Do you use glasses? Yes/No Do you know if you have myopia, hypermetropy or astigmatism? Do you know how much diopters? Do you have any ocular pathology? Yes/No Do you have any type of ocular treatment? Yes/No Do you have any type of ocular surgery performed? Yes/No

**Table 2:** Ophthalmological inclusion criteria.

Being free of ocular disease or posterior pole pathology Macular degeneration Drusen Glaucoma or suspicion Epiretinal membrane Congenital malformation Having a best corrected visual acuity more than 20/40. Having less than ± 5 spherocylindrical refractive error. Having intraocular pressure less than 20 mmHg.

**Table 3:** Demographic data of the study population.

	FH+ ApoE ɛ4 Carriers	FH− ApoE ɛ4 Non-Carriers	*p*-Value
Number of participants (*n*)	35	29	
Age (years)	57.00 (54.00–61.00)	59.00 (54.00–65.00)	0.164
Sex			0.028 *^,1^/0.353 ^1^
Male/Female	11/24	12/17	
MMSE	29.00 (29.00–29.00)	29.00 (28.00–29.00)	

Median (interquartile range); * *p* < 0.05. Mann–Whitney U and chi-square tests; ^1^ *p*-value of different sex in the same group; SD: s0tandard deviation; FH+: subjects with a family history of AD; FH−: subjects without a family history of AD. MMSE: Mini Mental State Examination.
